# TACC3-driven translation reprogramming dictates susceptibility or tolerance to mitotic stress

**DOI:** 10.64898/2026.09.18.752750

**Published:** 2026-09-22

**Authors:** Ozge Saatci, Aldo Hernández-Corchado, Adriana Aguilar-Mahecha, Breege Howley, Jennifer Rutherford Bethard, Jean-Sebastien Anoma, Josiane Lafleur, Marguerite Buchanan, Elizabeth G Hill, Lauren E Ball, Mathew Sajish, Philip Howe, Mark Basik, Hamed Najafabadi, Ozgur Sahin

## Abstract

Translation reprogramming is central to cancer cell plasticity under stress. However, the molecular players coordinating translation and epitranscriptomic rewiring to determine adaptive responses to mitotic stress remain elusive. Here, we found that microtubule targeting agent (MTA)-induced CDK1 blocks global translation while it phosphorylates and degrades TACC3, a multifunctional adaptor, releasing eIF4A/eIF4E/eIF4G2 initiation factors. This promotes selective m ^7^ G cap-dependent translation of mRNAs with ‘MTAup’ motif that are functionally required for apoptosis by disrupting proteostasis, promoting MTA-sensitivity. On the other hand, selectively translated TACC3 interacts with eIF3d/eIF4G1 and the m ^6^ A writer METTL3, mediating switch to m ^6^ A methylation and m ^7^ G cap-independent translation of mRNAs with hnRNPC-motif involved in chromosome segregation, driving MTA tolerance. TACC3 inhibition overcomes MTA resistance via restoring translation reprogramming. These findings demonstrate that TACC3 is a pivotal coordinator of translation/epitranscriptomic reprogramming and a therapeutic target in MTA-refractory cancers.

**Highlights:**

- MTA-induced CDK1 blocks global translation while increasing selective translation
- Cap-dependent translation of mRNAs with MTAup motif leads to MTA-induced apoptosis
- TACC3 fuels m ^6^ A methylation/switch to cap-independent translation and MTA tolerance
- TACC3 serves as a potential therapeutic target to overcome MTA resistance

## Full Text Availability

The license terms selected by the author(s) for this preprint version do not permit archiving in PMC. The full text is available from the preprint server.

